# An auditory-visual tradeoff in susceptibility to clutter

**DOI:** 10.1038/s41598-021-00328-0

**Published:** 2021-12-07

**Authors:** Min Zhang, Rachel N Denison, Denis G Pelli, Thuy Tien C Le, Antje Ihlefeld

**Affiliations:** 1 Department of Biomedical Engineering, New Jersey Institute of Technology, Newark, NJ USA; 2 Department of Biomedical Engineering, Rutgers New Jersey Medical School, Newark, NJ USA; 3 Department of Psychology, Boston University, Boston, MA USA; 4 Department of Psychology, New York University, New York, NY USA

**Keywords:** Human behaviour, Auditory system, Cognitive neuroscience, Sensory processing, Visual system

## Abstract

Sensory cortical mechanisms combine auditory or visual features into perceived objects. This is difficult in noisy or cluttered environments. Knowing that individuals vary greatly in their susceptibility to clutter, we wondered whether there might be a relation between an individual’s auditory and visual susceptibilities to clutter. In *auditory masking*, background sound makes spoken words unrecognizable. When masking arises due to interference at central auditory processing stages, beyond the cochlea, it is called *informational* masking. A strikingly similar phenomenon in vision, called *visual crowding*, occurs when nearby clutter makes a target object unrecognizable, despite being resolved at the retina. We here compare susceptibilities to auditory informational masking and visual crowding in the same participants. Surprisingly, across participants, we find a negative correlation (*R* = –0.7) between susceptibility to informational masking and crowding: Participants who have low susceptibility to auditory clutter tend to have high susceptibility to visual clutter, and vice versa. This reveals a tradeoff in the brain between auditory and visual processing.

## Introduction

Hearing-impaired individuals often misunderstand speech in the presence of background sound. Typically this occurs because the same neurons in the auditory nerve respond to both the target of interest and the background sound, swamping target information, a phenomenon known as *energetic masking*. An extensive literature reveals auditory band-pass filters (or “critical bands”) across the spectral range of the cochlea^1^, but see also^2^ Briefly, the signal detection threshold in notched noise as a function of notch width traces a tuning function shaped like a *ro*lling *ex*ponential, or *roex*, function^3^. The signal-to-noise ratio (SNR) within the equivalent rectangular bandwidth (ERB) of these roex functions accurately predicts how well an individual understands spoken words in background noise^4^. However, the critical-band SNR fails to predict the drop in speech intelligibility that occurs when the background sound is made perceptually similar to the target sound. Speech is unintelligible when masked by noise at the speech frequencies, and clear when the noise spectrum is non-overlapping. However, unlike that noise, a background sound that is perceptually similar to the target can make words unintelligible even when its spectrum is non-overlapping^5^. This second difficulty with background sound is attributed to the central auditory phenomenon called *Informational Masking (IM)*^6^.

**Figure 1:**
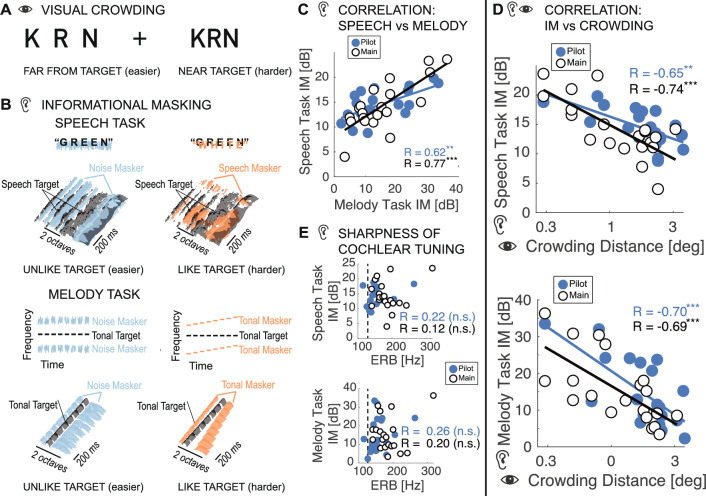
Blue and white symbols represent the pilot and main experiments, respectively. (**A**) To experience crowding, fixate the cross. While fixating the cross, try to identify the middle letter in each triplet (left and right), ignoring the outer letters. When the spacing is tighter, the target is harder to identify. Without the outer letters, the two targets, left and right, would be equally legible. *Crowding distance* is the center-to-center target-flanker separation (in visual degrees) required to attain 70% correct recognition of the target. (**B**) Schematics and spectrograms of the sounds we used to study informational masking (IM). The noise masker covered the same spectral range as the target-like masker, so they excite comparable regions in the cochlea. Thus, any excess masking by an equal-energy target-like masker is post-cochlear, i.e., IM. We measure the participant’s accuracy in identifying the target sound (denoted in black) as a function of the *Target-to-Masker broadband energy Ratio* (TMR). The target is more easily identified when the masker is unlike the target (left spectra), so threshold TMR for (target-unlike) noise masking is lower than that for target-like masking. *IM susceptibility* is the difference in threshold TMRs between the target-like and (target-unlike) noise backgrounds. (**C**) Participants who are IM-susceptible in the speech task also tend to be IM-susceptible in the melody task. (**D**) However, IM susceptibility in both the speech (upper graph) and melody (lower graph) tasks is anti-correlated with the participant’s crowding susceptibility. (**E**) Both the speech and melody graphs show that the Equivalent Rectangular Bandwidth (ERB) is a poor predictor of IM susceptibility. To rule out energetic masking as a trivial explanation of our results, we confirmed that our participants had appropriate cochlear tuning. Specifically, ERBs at the target center frequency of 1000 Hz, estimated from noise-masking thresholds in the melody task, were generally smaller than the smallest tested notch width. Note that a 0.3-octave notch width corresponds to 208 Hz at 1000 Hz. One participant’s ERB exceeded 208 Hz, but removing this participant does not affect the conclusions. No normative data exist for normally hearing ERBs with the specific stimuli used here. However, for approximate comparison, the vertical black dashed line denotes the ERB one would expect to see in normal-hearing listeners for a single-tone target embedded in notched noise^1^. Note, to eliminate distortion product cues from interactions of target and melody masker tones, we here added low-intensity broadband white noise, widening the observed ERBs.

IM is defined purely phenomenologically. It occurs when target and masker resemble each other or when the listener is uncertain about their perceptual properties^7,8^. The development of assistive listening devices that could compensate for IM is challenged by the great variation in susceptibility to IM across individuals^9^. The source of this individual variability is poorly understood. Curious about the origin of the variability, we here compare IM to a phenomenologically similar effect in vision: crowding^10^.

Vision and hearing are very different. For instance, comparing sensory organs, the retina is a spatial analyzer, whereas the cochlea is a frequency analyzer. Despite being in such different senses, IM and crowding are strikingly analogous. Both phenomena are cortical failures to recognize a target in target-like clutter^11–16^. Both are resistant to learning^17,18^. In crowding, nearby clutter is perceived as part of the visual target (Fig. 1A). Analogously, in IM, flanking sound, with little energy inside the target’s critical band, hampers individuation of the target (Fig. 1B). Crowding and IM are stronger when target and clutter are perceptually similar. Both phenomena occur even when the clutter is much fainter than the target, or when the target is presented to one eye or ear and the clutter to the other eye or ear^6,10,19–21^. Given these functional similarities, we wondered whether IM and crowding rely on similar mechanisms.

In vision, clutter crowds a target, preventing recognition, when the clutter is too close to the target. We measure the *crowding distance* (a.k.a. critical spacing), namely the required spacing to achieve 70%-correct recognition of the target. Crowding distance predicts an individual’s crowding susceptibility over a broad range of conditions^10^. In hearing, background sound informationally masks a target that is too perceptually similar to the target. We measure IM susceptibility, namely the change in Target-to-Masker broadband energy Ratio (TMR) at perceptual threshold between target-like vs. target-unlike masking sound. Here we test the hypothesis that susceptibility to clutter is not specific to just hearing or vision and that susceptibility to visual clutter predicts susceptibility to auditory clutter.

## Results

We measured behavioral performance in one crowding and two IM tasks in the same 20 normal-hearing young participants. To measure crowding distance, participants identified a peripheral target letter between two flanking letters (Fig. 1A). To quantify IM, we used a speech and a non-speech task. In both tasks, we separately tested maskers that were either target-like or noise, which is not target-like, and varied the TMR. IM susceptibility is the difference between threshold TMRs in target-like background sound vs noise. In the “speech task,” participants identified target words constructed from narrow spectral bands that were masked by either speech (target-like) or noise. In the “melody task”, participants reported whether a target sequence of eight constant frequency tones was present while spectrally flanked by masking sequences of either target-like tones or noise bursts (Fig. 1B).

We initially ran this experiment as a pilot study with 20 participants. Prompted by the unexpected findings, we re-ran it as the “main” experiment with a new group of 20 participants and minor modifications (see Methods). Results of the pilot and main experiments are similar (compare blue vs white symbols in Fig. 1C-E). IM susceptibility in the melody task predicts IM susceptibility in the speech task (pilot: $R=0.62$, $p=0.003$; main: $R=0.77$, $p<0.001$, Fig. 1C), indicating that IM susceptibility generalizes across speech and non-speech stimuli. Moreover, across participants, crowding susceptibility (i.e. crowding distance) and IM susceptibility are correlated, but, unexpectedly, the correlation is negative. In this way, crowding is inversely related to IM for both the speech task (pilot: $R=-0.65$, $p=0.002$; main: $R=-0.74$, $p<0.001$) and the melody task (pilot: $R=-0.70$, $p<0.001$; main: $R=-0.69$, $p<0.001$, Fig. 1D). Equivalent rectangular bandwidth (ERB), estimated from noise masking thresholds, does not correlate with IM susceptibility (Speech: pilot: $R=0.22$, $p=0.4$; main: $R=0.12$, $p=0.6$; Melody: pilot: $R=0.26$, $p=0.261$; main: $R=0.20$, $p=0.399$, Fig. 1E), confirming that an individual’s sharpness of cochlear tuning does not predict their IM susceptibility^22^.

## Discussion

IM and crowding are analogous perceptual processes in hearing and vision. We find that they are inversely related. People with high tolerance for visual clutter tend to have low tolerance for auditory clutter, and vice versa. This is true for both speech and non-speech sounds.

The neural origin of the observed tradeoff between auditory and visual processing is unclear. Common mechanisms cannot explain this effect as they should induce positive correlations between IM and crowding. For instance, selective attention, motivation, effort, and vigilance modulate susceptibility to IM^23,24^. However, there typically is either no association across selective attention, motivation, effort, and vigilance in unimodal hearing or vision tasks, or people who are good at tracking objects with their ears are also good at tracking **them with their eyes^25^, thus ruling out domain-general cognitive processing as an explanation for the observed anticorrelation. Another potential caveat, developmental deprivation through childhood undernourishment, can lead to cognitive impairment, but there is no evidence that it differentially shapes hearing vs. vision^26^.

IM and crowding cannot be accounted for by models of cochlear and retinal processing. There is strong evidence that they are central. IM operates on auditory objects, which emerge in auditory cortex^27^. Moreover, susceptibility to IM is inversely related to recruitment of auditory cortex^28^. Crowding is strongly affected by similarity of orientation^29,30^, and visual cortex is the first stage of visual processing that shows orientation tuning^31^. Information from the two eyes is first combined in visual cortex, so crowding produced by dichoptic presentation of the mask to one eye and the signal to the other shows that crowding is a cortical, not a retinal phenomenon^21,32,33^.

Primary visual cortex (V1) and primary auditory cortex (A1) tend to covary in size^34^, which would suggest a positive association between visual and auditory performance. However, crowding distance correlates with size of hV4, not V1, V2, or V3^35^, and the size correlation between A1 and hV4 is unknown. Visual areas V1, V2, V3, and hV4 are in posterior cortex, whereas auditory cortex is more anterior, in the superior temporal gyrus. Patients with posterior cortical atrophy are more susceptible to visual crowding and—surprisingly—less able to perceptually segregate auditory scenes^36^, unlike our negative correlation in neurotypical participants.

Finally, we wonder if years of prolonged visual or auditory attention, e.g. by an artist or musician, might reduce crowding or IM, respectively. People who spend more time looking may listen less, and vice versa. Foveal crowding distance drops 3-fold from age 3 to 8, not reaching the adult level, but we don’t know whether this drop reflects experience or maturation^37^. IM takes longer to mature, reaching adult levels until the early teenage years^38^. Further work is needed to discover how an individual’s ability to recognize a target in clutter develops in each sensory modality.

## Methods

### Participants

A total of 40 participants (ages 19–25) took part in the study, 20 per experiment (8 females in the main experiment, and 10 females in the pilot). All participants had normal audiometric pure-tone detection thresholds as assessed through standard audiometric testing at all octave frequencies from 250 Hz to 8 kHz. At each tested frequency, tone detection thresholds did not differ by more than 10 dB across ears, and all thresholds were 20 dB HL or better. All participants self-reported that they had never learned to play an instrument and never sung in a vocal ensemble. All participants gave written informed consent to participate in the study. All testing was approved by the Institutional Review Board of the New Jersey Institute of Technology. All experiments were performed in accordance with relevant guidelines and regulations.

### Experimental setup

Throughout testing, participants were seated inside a single-walled sound-attenuating chamber (International Acoustic Company, Inc.) with a quiet background sound level of less than 13 dBA. Acoustic stimuli were generated digitally (32 bit resolution, 44.1 kHz sampling frequency) in MATLAB (Release R2016a, The Mathworks, Inc., Natick, MA, USA), converted to analog by a sound card (Emotiva Stealth DC-1; Emotiva Audio Corporation, Franklin, TN, USA), and presented over insert earphones (ER-2, Etymotic Research Company Elk Grove Village, IL, USA). The acoustic setup was calibrated with a 2-cc coupler, 1/2” pressure-field microphone, and a sound level meter ( 2250-G4, Brüel and Kjær, Nærum, Denmark). Visual stimuli were delivered via a 23-inch monitor with 1920 x 1080 resolution. Prior to visual testing, the experimenter positioned the monitor such that it was centered 50 cm away from the center of the participant’s nose. Using this setup, three task were administered in an order that was counterbalanced across participants.

**Figure 2:**
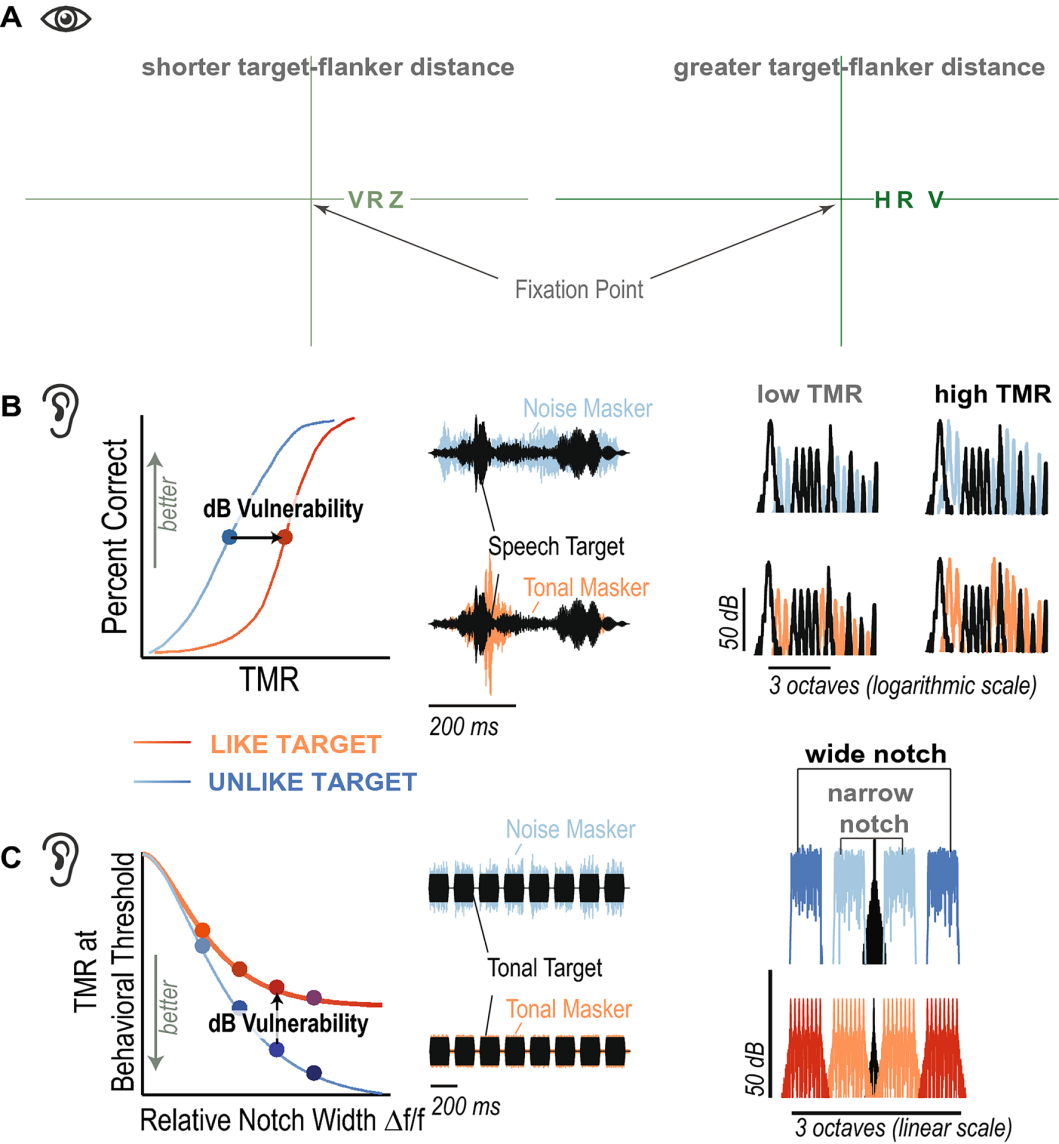
**(A)** In the visual crowding task, participants fixated the cross and called out the middle letter, i.e. the target letter (here: *R*), surrounded by flanking letters that acted as clutter. Crowding distance was measured by adaptively varying the center-to-center spacing of the target and flankers^39^. In the example here, the target is 10 deg right of the fixation cross. In the main experiment, the target would appear randomly 10 deg to the left or right of the cross, whereas in the pilot experiment it appeared only to the right. **(B)** In the speech task, susceptibility to IM was measured as the difference between the TMR at which participants correctly identified 50% of target words in the presence of background speech vs. that in background noise. **(C)** Analogously, in the melody task, susceptibility to IM was assessed as the difference in TMRs between target detection with melody maskers vs. noise maskers, and across different notch widths.

### Visual crowding task

Crowding distance was measured with a target identification paradigm^39^, illustrated in Fig. 2A. Participants were instructed to fix their gaze on the center of a cross hair displayed on the monitor in front of them. A target letter was briefly displayed, 10 deg from fixation along the horizontal midline. In the pilot experiment the target was always to the right of fixation; in the main experiment the target was randomly left or right of fixation. (Randomly interleaving trials with the target on right or left is meant to minimize the incidence of anticipatory movement of gaze away from fixation toward the expected target location, by producing presumably equal and opposite leftward and rightward urges that cancel each other out.) The target letter was shown between two flanking letters, as a row of three black letters in the Sloan font. As explained below, the center-to-center spacing of the three letters changed from trial to trial. The size of the (nearly square) letters was always the letter spacing divided by 1.4. The three letters were randomly taken, without replacement, from nine possible letters: D, H, K, N, O, R, S, V, Z. The target and flankers appeared for 200 ms, and then disappeared. Participants were asked to read the middle letter out loud, while maintaining fixation on the cross hair. An experimenter recorded the participant’s verbal response on each trial.

In all our crowding tests, the three letters were spaced unequally in order to achieve equal spacing on the visual cortex. The unequal spacing of the three letters (e.g. V R Z) may be apparent in Fig. 2A. The inner (closer to fixation) flanker is spaced at the nominal spacing from the target. The outer flanker is farther. Its spacing is computed to equate spacing of the three letters on the surface of the visual cortex, assuming that position (mm) on the cortex is proportional to log radial eccentricity (deg)^15^.

For each participant, in both pilot and main experiments, our subsequent analysis is based on a pair of thresholds (i.e. crowding distances). The pair of threshold measurements differed between the pilot and main experiments. The pilot measured the right-side threshold twice in succession. The main experiment made randomly interleaved measurements of the left- and right-side thresholds. In the pilot, for each participant, we measured the two thresholds, and, if they differed by more than 0.2 deg (true for 11 out of the 20 observers), we measured them again. In the main experiment we always measured them twice. The repeated measurement helped, presumably by giving the participants practice. In the first pair of thresholds the standard deviation of the difference was 0.9 in the pilot and 0.4 in the main experiment. In the second pair of thresholds that standard deviation was only 0.1 in both the pilot (for the 11 of 20 that did it) and the main experiment (for all 20). For the pilot, the standard deviation of the difference between the last two measurements was also 0.1 (for all 20). In the pilot experiment we used only the single last-measured threshold. In the main experiment we used the average of the last-measured left and right thresholds. For pilot and main experiments, those are the crowding distances reported throughout this paper.

Every staircase had 40 trials and was independently guided by the QUEST algorithm^40^ to efficiently estimate the crowding distance, i.e. the 70%-correct threshold center-to-center target-flanker spacing. Each staircase was primed with an initial guess for distance from the center of the middle letter based on neurotypical critical spacing^41^.

### Speech task

IM susceptibility was measured in a speech task (Fig. 2B), by subtracting the TMR at 50% correct speech identification threshold with a speech masker from the threshold TMR with a noise masker^42^. Speech identification was assessed using the Coordinate Response Measure matrix task^43^. This matrix task uses sentences of the following fixed structure: ’Ready [callsign] go to [color] [number] now.’ During testing, a target and a masker sentence were simultaneously presented to the left ear only, constrained to differ from each other in terms of callsign, color and number keywords^44^. Target sentences always had the callsign ’Baron.’ There were four color keywords $<\hbox {'red','blue','white','green'}>$ and seven possible numbers $<\hbox {'one','two','three','four','five','six','eight'}>$ (excluding the number ’seven’ because it has two syllables. Participants were instructed to answer the question: ’Where did Baron go?’ by pressing the corresponding color and number buttons on a touch screen response interface. A trial was counted as correct if the participant correctly reported the target color and the target number, resulting in a chance performance of 4% ( $\frac{1}{4}\cdot \frac{1}{7}=\frac{1}{28}=0.04$).

To vocode the utterances, raw speech recordings were normalized in root mean square (RMS) value and filtered into 16 sharply tuned adjacent frequency bands using time reversal filtering, resulting in no appreciable phase shift. Each resulting band covered 0.37 mm along the cochlea between 3-dB down-points according to Greenwood’s function^45^, or approximately 1/10th octave bandwidth, and had a 72 dB/octave frequency roll-off, with center frequencies ranging from 300 to 10,000 Hz. In each narrow speech band, the temporal envelope of that band was then extracted using the Hilbert transform and multiplied by uniformly distributed white noise carriers. To remove the side bands, the resulting amplitude-modulated noises were processed by the same sharply tuned filters that were used in the initial processing stage. Depending on the experimental condition, a subset of these sixteen bands was then added, generating intelligible, spectrally sparse, vocoded speech. Stimuli were generated from utterances by two different male talkers, one for the target and a different talker for the speech masker.

To generate noise maskers that matched the spectrum of the vocoded speech, all processing steps were the same as in the vocoding described above, with one exception. Instead of using the Hilbert envelope to amplitude-modulate the noise carriers, here, the noise carriers were gated on and off with 10 ms cosine-squared ramps that had the same RMS as the Hilbert envelope of the corresponding speech token in that band. A subset of the resulting 16 narrowband pulsed noise sequences was added to generate low-IM noise maskers.

On each trial, nine randomly chosen bands were added to create the target. The masker was comprised of the remaining seven bands and either consisted of vocoded utterances from the same corpus, recorded by a different male talker (target-like) or of noise tokens with similar long-term spectral energy as the vocoded utterances (target-unlike). The center and right panels of Fig. 2B shows a representative temporal and spectral energy profile for a mixture of target (black) and speech (purple) or noise (brown) maskers.

The masker was presented at a fixed level of 55 dB SPL. The target level varied randomly from trial from 35 to 75 dB SPL, with a 10 dB step size, resulting in five broadband TMRs from -20 dB to 20 dB. For familiarization with the vocoded speech task and to ensure that the vocoded speech stimuli were indeed intelligible, participants were initially tested in 20 trials on nine-band target speech at 35 dB SPL, without masking. Target bands varied randomly from trial to trial. All participants reached at least 90% accuracy during this testing in quiet.

Next, participants were tested in five blocks of 40 trials while target-unlike noise or target-like speech interfered in the background. Thus, each specific combination of the five different TMRs and two masker types was presented 20 times (5 TMRs * 2 masker types *20 trials = 5 blocks *40 trials = 200 trials total). TMR and masker type varied randomly from trial to trial such that all combinations of TMR masker type were presented in random order once before all of them were repeated in a different random order.

To estimate the TMR at the 50% correct threshold for each participant, percent correct scores as a function of TMR were fitted with Weibull-distributed psychometric functions, by using the psignifit package^46^. IM susceptibility was computed as the difference in TMR at 50% correct between noise vs. speech masking.

### Melody task

IM susceptibility was also assessed using two-up-one-down adaptive tracking with a non-speech task, by contrasting 70.7% correct thresholds for detecting eight-tone-burst targets across two notched-masker conditions: a noise vs. a melody masker^47^, illustrated in Fig. 2C. Target and masker were presented to the left ear only. The target consisted of eight pure tones at a fixed frequency of 1000 Hz. Each tone was 150 ms long (including 10 ms cosine-squared ramps, random phase), with 75 ms gaps between consecutive tones. The target intensity was varied adaptively.

Using a classic paradigm for estimating ERB, in the noise masker condition, two 600-Hz-wide narrow bands of noise were placed symmetrically around the target frequency, creating a symmetrical notch in logarithmic frequency^3^. The notch width was one of the following: $<0.3, 0.5, 1, 1.5>$ octaves. Noise tokens with very steep spectral slopes of over 400 dB/octave were constructed by generating uniformly distributed white noise, transforming it via Fast Fourier Transform and setting the notch frequencies in the spectrum to 0, before transforming the signal via the real portion of the inverse Fast Fourier Transform back into the time domain.

The melody masker condition was designed to closely match the spectral profile of the noise masker. Two eight-tone melodies, each carrying eight possible frequencies that were spaced linearly within 600-Hz-wide bands, flanked the target. One of these melodies was played above, the other below the target frequency, positioned symmetrically around the target frequency along a logarithmic frequency axis. The maskers were chosen from four possible melodies $<\hbox {up}, \hbox {down}, \hbox {up-down}, \hbox {down-up}>$. Those patterns indicated how the frequency changed for eight pure tones that formed the sequence, for instance ’up’ means that each pure tone increased in frequency compared to the previous one in the sequence. The phase of each tone was independently and randomly drawn for each tone, resulting in phases that generally differed across all tones in the target-flanker mixture.

Maskers were played at a fixed spectrum level of 40 dB SPL, equivalent to a broadband level of 68 dB SPL (total level = $40+10\cdot log_{10}(300) + 10\cdot log_{10}(2) =68$). To protect against the possibility of distortion products as a possible task cue in the melody condition, a low-intensity broadband white noise masker was continuously played in the background during both the noise and the melody masker condition at 15 dB SPL.

Under both masker conditions, participants performed a two-alternative forced-choice target detection task, responding with ’yes’ or ’no’ to indicate whether they heard the target. At the beginning of each adaptive track, the target intensity started at 70 dB SPL. The target intensity was initially decreased by 10 dB for every two consecutive correct answers and increase by 10 dB for every incorrect answer. When the slope in target intensity changed from decreasing to increasing after an incorrect response, the trial was called a *reversal*. After every two reversals in the adaptive tracks, the step size was halved. Participants completed 12 reversals, resulting in approximately 80 trials per participant and adaptive track. The threshold was the average target intensity across the final 12 trials. IM susceptibility was calculated by subtracting thresholds between noise and melody masker at one octave separation.

### Equivalent rectangular bandwidth (ERB)

To estimate each participant’s ERB, noise masked thresholds from the melody task, denoted as *W*, were minimum-least-square fitted to rounded exponential (roex) functions (command lsqcurvefit in MATLAB). The roex functions were defined as $W(g)=(1-r)(1+pg) e^{-pg}+r$, where *p* determined the steepness of the roex function’s passband, *r* shaped the stopband, and *g* denoted the distance between the target frequency $f_T$ and the corner frequencies of the masker notch with $g = \frac{f-f_T}{f_T}$^22,48^.

### Pilot experiment

The methods used for the pilot experiment were similar to those of the main experiments except for two differences. First, unlike in the main experiments, the tasks in the pilot experiment were not counterbalanced across participants and administered in fixed order instead. Specifically, during pilot testing, participants first completed the crowding task, followed by the IM speech task, followed by the IM melody task. The second difference across the experiments is that in the crowding task during piloting, we only measured crowding distance at one location (to the right of fixation), and the target always appeared there. Whereas during the main experiments, we measured crowding distance at two locations (to the right and left of fixation), and, since the conditions were interleaved, the target appeared unpredictably to the left or right of fixation.

### Statistical analysis

Statistical analyses were performed using linear regression via the command fitlm in MATLAB 2019b, and *R* and adjusted *R*-squared from these fits are reported. Multiple comparisons were adjusted with Bonferroni correction.

## Supplementary Information

Supplementary Information.
