# Supplementary material for: An auditory-visual tradeoff in susceptibility to clutter
Source: Sci Rep. 2021 Dec 7;11:23540. doi: 10.1038/s41598-021-00328-0 (PMC8651672; doi:10.1038/s41598-021-00328-0)
Supplement: Supplementary file 1 — Supplementary Information. [file 41598_2021_328_MOESM1_ESM.pdf]

## Supplemental Information: Difference between pilot and main participants

We recruited the participants for the pilot and main experiments in the same way approximately a year apart, and tested them using identical equipment, but, on average, participants in the pilot experiment were more susceptible to visual crowding though equally susceptible to auditory clutter. The average crowding distance for a Sloan-font target 10 deg right of fixation was $2.0 \pm 0.2$ deg in the pilot and $1.4 \pm 0.2$ deg in the main experiment (mean $\pm$ se, $N = 20$). That main-experiment result agrees perfectly with the finding of a larger study of crowding in 78 participants ($1.4 \pm 0.1$ deg crowding distance for a Sloan target 10 deg right of fixation, Dataset A in Table 4 in[35]). While worse with visual clutter, the pilot participants, on average, did about as well as the main participants with auditory clutter (Speech IM: pilot $14.5 \pm 0.7$ dB vs. main $14.3 \pm 1.1$ dB; Melody IM: pilot $14.9 \pm 2.1$ dB vs. main $15.5 \pm 2.0$ dB). The visual difference between the pilot and main participants is noticeable in Figure 1D. The blue points represent the pilot participants and the white points represent the main participants. The two sets of points have similar regression lines, but there are fewer blue than white dots at smaller crowding distances. Thus, on average, the pilot participants were more susceptible to visual clutter, but the two groups have similar regression lines and audio-visual tradeoff in susceptibility to clutter.

## Supplemental Information: Melody task

The majority of our participants had ERBs at or below 208 Hz (Figure S.1A), with no appreciable differences between pilot and main participant groups in the sharpness of cochlear tuning. As expected, thresholds were much less variable across listeners in the the noise masker condition than in the melody condition, in both the main and pilot experiments (compare the spread in the density plots in the top vs. bottom panels of Figure S.1B). Given the high variability across participants of IM-susceptibility, we made bootstrapped estimates the confidence interval of the correlation between crowding distance and susceptibility to non-speech IM, as a function of notch width (Figure S.1B). Adjusted correlation coefficients roughly increased with increasing notch width and were most consistent across the main vs. pilot experiments at the 1-octave notch width (Figure S.1C).

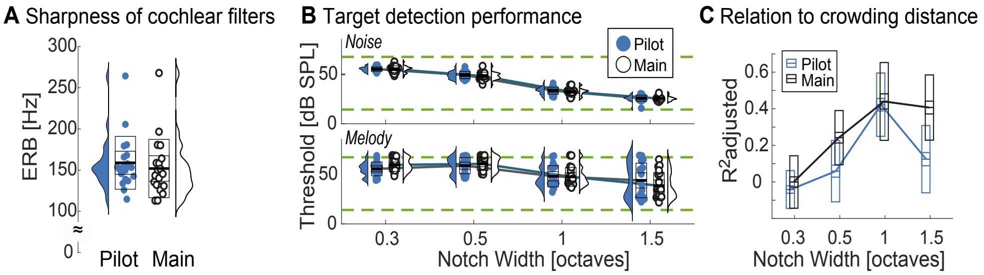


**Figure S.1.** **(A)** The range of ERBs is similar across the two experiments. **(B)** Individual variability is much higher in target-like masking than noise across all tested notch widths. For all participants, target detection thresholds generally fall between the broadband level of the notched masker (68 dB SPL) and broadband masker (15 dB SPL), shown by green dashed lines. In the noise masker configuration, target detection thresholds decrease with increasing notch width for all participants (top), but only for some participants in the melody masker configuration (bottom). Note that in both the main and the pilot experiment, the probability density of data points in the melody task is bimodal, with one mode close to 68 dB SPL throughout, and the other mode decreasing with increasing notch width. **(C)** The adjusted correlation coefficient between visual crowding distance and IM susceptibility reveals coarse tuning. Results between the two experiments are congruent at 0.3 and 1 octave notch widths, but appear to diverge at 0.5 and 1.5 octave notch widths. Test-retest variability of $R^2$, estimated via bootstrapping that sampled 10 out of 20 participants without replacement 100 times, shows that, indeed, crowding distance is robustly correlated with IM susceptibility at 1 octave separation, in both experiments. At other notch widths, the relationship is less pronounced.

Visual inspection of the probability density functions in Figure S.1B hints that the distribution of melody masking thresholds was bimodal, gradually widening with increasing octave separation. The mean of the lower mode, corresponding to participants who were more resilient to masking, decreased with increasing notch width. The mean of the other mode remained roughly constant as a function of notch width and close to the broadband level of the masker, indicating that the more poorly performing participants chose a strategy to listen for the louder source as opposed to relying on target pitch. In these poorly performing listeners, thresholds did not monotonically improve with increasing notch width. Perhaps as a result, roex functions used to estimate ERB under noise masking did not provide appropriate fits of the data under melody masking.

While we did not originally anticipate this result, in general, approximately a third of normal-hearing listeners have difficulty discerning pitch, and can, for instance, not reliably distinguish between major and minor triads in musical chords, even when given trial-by-trial correct response feedback.[49–51] Note that we here tested the IM melody task at 0.3, 0.5, 1 and 1.5 octave

notch width, resulting in center frequencies of the lower and upper flanker bands that were related by factors of 1.231, 1.414, 2.000 and 2.828. Those numbers were originally chosen to cover the range of notch widths that typically result in ERB estimates.[48] However, they had the unintended effect that the constituent flanker frequencies were not perfectly harmonically related, and therefore potentially unfused at 0.3, 0.5 and 1.5 octaves, whereas flanker frequencies were harmonically related at the 1 octave notch. In summary, IM susceptibility in the melody task at these three other notch widths is more weakly correlated or even uncorrelated with crowding distance (as well as IM susceptibility to speech), showing that harmonicity affects IM in this paradigm. Moreover, domain-general selective attention[25] or systemic developmental deprivation[26] neither accounts for this $R^2$-tuning nor for the inverse association between IM and crowding in the main experiment.
